# Study of the soluble salts formation in a recently restored house of Pompeii by in-situ Raman spectroscopy

**DOI:** 10.1038/s41598-018-19485-w

**Published:** 2018-01-25

**Authors:** Nagore Prieto-Taboada, Silvia Fdez-Ortiz de Vallejuelo, Marco Veneranda, Iker Marcaida, Héctor Morillas, Maite Maguregui, Kepa Castro, Ernesto De Carolis, Massimo Osanna, Juan Manuel Madariaga

**Affiliations:** 1 Department of Analytical Chemistry, Faculty of Science and Technology, University of the Basque Country, UPV/EHU, Barrio Sarriena s/n, 48940 Leioa, Spain; 2 Department of Analytical Chemistry, Faculty of Pharmacy, University of the Basque Country, UPV/EHU, PO Box 450, 01080 Vitoria-Gasteiz, Spain; 3 Archaeological Park of Pompeii, via Villa dei Misteri 2, 80045 Pompeii, Italy; 4 Unesco Chair on Cultural Landscapes and Heritage, University of the Basque Country, UPV/EHU, P.O. Box 450, 01006 Vitoria-Gasteiz, Spain

## Abstract

The walls and mural paintings of Pompeii exposed directly to the rainfalls are the most impacted in view of the observed decay. However, there are also wall paintings in protected rooms showing evidences of decaying. The aim of this research was to study the salts formed in such protected wall paintings only by non-invasive and *in-situ* Raman spectroscopy to understand their decaying processes. The perystile of the House of the Gilded Cupids (Regio VI, Insula 16), one of the most important houses of Pompeii was studied. Although an exhaustive restoration was carried out in 2004, a new conservation treatment was needed in 2013 and only two years later, extensive crystallizations of soluble salts were again threatening several of the restored surfaces, thus, the presence of an unsolved degradation pathway was deduced. Thank to the proposed methodology, it was pointed out that the key is the acidified rainfall impact in the non-protected backside of the walls containing the wall paintings. Thus, a new concept in the preservation of the houses of Pompeii is provided, in which the need of the protection of those walls from both sides is suggested to avoid the movement of water through the pores of the walls.

## Introduction

Pompeii is one of the most important archaeological sites in the world, where it is possible to travel through time and observe the magnificence of the ancient Rome. However, due to the exceptional size of the ancient city of Pompeii and the particular characteristics of this archeological site (volcanic eruptions, atmospheric acid gases attack, etc.), its decaying could be inevitable, leading to the loss of artworks of inestimable cultural and historical value. In order to avoid it, the direction of the Archaeological Park of Pompeii is carrying out intense rehabilitation works and promoting different scientific researches with a common target: stop the decaying processes and improve the long-term restoration works^1^. Since 2012, activities of protection and enhancement have been further stimulated by the “Grande Progetto Pompei”, founded by the European Union^1^.

The Analytica Pompeiana Universitatis Vasconicae (APUV) project, an initiative from the IBeA research group of excellence belonging to the University of the Basque Country (UPV/EHU) in collaboration with the direction of the Archaeological Park of Pompeii, started some years ago aiming to understand the reasons for the decaying of the frescoes and building materials of some of the most important Pompeian houses^2^. Under APUV project and in order to achieve the mentioned purpose, different research expeditions since 2010 have been carried out and thanks to them, IBeA has acquired an important background. The reasons/causes of the observed decaying processes are complex, but the APUV expeditions pointed out the effect of environmental factors as one of the most important item for the conservation state of this archaeological site. Ancient aggressive atmosphere from volcanic eruptions, the modern and polluted atmosphere and meteorological factors such as rain water are some of the causes that can promote the decaying of the construction materials used to build the wonderful houses of Pompeii^3–6^.

Focusing on the decaying processes, the formation of soluble salts is one of the most damaging pathways, which can irreversibly harm frescoes and walls. Previous research works of the APUV project^7^ and other authors^8,9^ have related these formations with the interaction between the ions contained in the water (direct contact, infiltration or capillarity phenomena, ground water, rainfall, etc.) and construction materials of the walls. Roofs are commonly used to protect the wall paintings that could be affected by rainfalls. However, there are several cases in which extensive crystallizations of soluble salts are observable on mortars and wall paintings sheltered by large roofs. This is the case of the House of Gilded Cupids, a very important house of Pompeii, restored the last time in 2013, which presents a considerable affection by soluble salts in the walls of the peristyle that surround the central garden. These walls are well protected with a large modern wooden roof erected in 2004. Thus, the formation of (sub)efflorescences after a specific restoration, conducted to remove the salts from these wall paintings, is not understood and it is against the conclusions obtained in the last researches^7^. Hence, to study the processes for the formation of these salts it seem to be crucial the understanding of why well protected walls are affected by this problem. This knowledge will aid to propose effective long-term restoration solutions.

To explain this unexpected behavior of some walls protected by roofs, chemical analysis of the efflorescence salts became mandatory in order to identify the most probable decaying mechanisms and therefore, to try to stop them. The analysis of the soluble salts can be carried out either by spectroscopic or chromatographic techniques^8–11^. However, as sampling is forbidden in this house, such analysis must be performed only using non-invasive portable instrumentation. Moreover, it has been demonstrated the salts formation is a very unstable phenomena because some hydrated salts are dependent on environmental conditions leading to different compounds during the *in-situ* and the laboratory analyses^12^. Raman spectroscopy gives high quality molecular information, even polymorphic, it does not require sampling, it is non-destructive, the information is obtained in real time and, sometimes, it is not required the support of complementary techniques, among other advantages^7,8,11,13^. Moreover, this technique has shown a constant technological development in the last years achieving very reliable portable equipments with good signal-to-noise ratio, even comparable to the laboratory instruments^14–16^.

Taking all of these into account, this work has two main objectives. On one hand, this work aims to study the salts formed in the protected wall paintings with different orientations from the peristyle of the House of Gilded Cupids by using only portable Raman spectroscopy, as well as to correlate them with their possible sources, to understand the reason for their formation and to assess the risk of deteriorated wall paintings by soluble salts and propose a solution. On the other hand, the present work also aims to point out how *in-situ* Raman spectroscopy is one of the best analytical techniques for the analysis of soluble salts in cultural heritage in a fast and reliable way and without required the support of other techniques or take samples, even to take relevant decisions in conservation works showing the most adequate practices for this spectroscopic technique.

## Experimental

### House of Gilded Cupids

**Figure 1:**
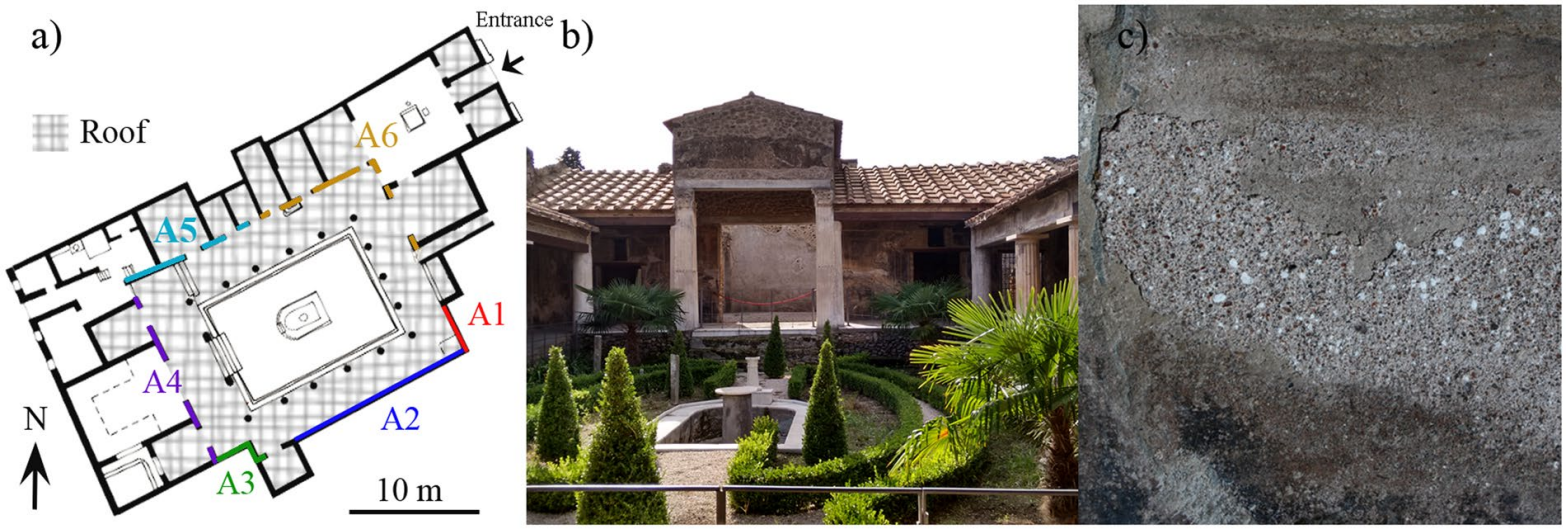
(**a**) Plan of the House of Gilded Cupid (Pompeii) showing the different measured areas (A1-A6). (**b**) Recent photograph of the wooden roof dated from 2004 that covers the wall paintings of the peristyle. (**c**) Detail of the sub-efflorescences formed in the intonaco in one of the studied walls.

The studied house is situated on the Via del Vesuvio (Reg VI, Insula 16, 7). Its name comes from some decorations, with cupids representations made of gold, found in the house and nowadays stored in the Naples National Archeological Museum (MANN). The owner seems to be related to Poppea Sabina, Nero’s second wife, thus, the house is a refined dwelling and richly decorated throughout as might be expected^1^. Mythological subjects and landscapes of the 3^rd^ style adorn the walls of the house, containing also paintings of 1^st^ and 4^th^ styles. The house has an amazing garden decorated with reliefs and sculptures of marble in the center of the peristyle (Fig. 1a,b). Moreover, the peristyle presents a typical roman lararium for traditional worship made of masonry and another one, more atypical, to adore Egyptian deities as a wall painting. In addition, in the walls of the peristyle, the unique glass mirror preserved in the whole city can be found.

Thanks to a restoration from 2004, a wooden ceiling protects the 4^th^ style frescoes of the peristyle walls (Fig. 1b). However, the conservation state of some wall paintings and plaster decorations is poor (see Fig. 1c) showing how the protection of the roofs is not enough to guarantee a good preservation of the artifacts contained in the house. Thus, the need to study more in depth the causes of deterioration is evident.

The sampling was forbidden in order to preserve the integrity of the house. In this sense, the analytical study had to be carried out by *in-situ* Raman Spectroscopy which allowed the measurement without take samples. For this purpose, the first step was selecting the areas for their study. Taking into account that the main problem was located in the peristyle, all the walls that form it were selected. All of them are covered by roofs thus none is supposed to be affected by direct rainfall impact on its front, in contrast to a previous study conducted on open air walls in the House of Marcus Lucretius^7^. However, some of the walls have different situations affecting their back sides, and also different level of decaying. For that reason the walls were divided in six different areas (A1-A6). Regarding their backsides, the A1 has a garden of the neighbor house. The backsides of the A2 and A3 walls belong to open air rooms of the same neighbor house, without roof protection. The backside of the A4-A6 walls belong to rooms of the House of Gilded Cupid, however, the A4 backside is an open air room and the rooms behind the A5-A6 walls are protected by roofs.

In the case of the observed decaying, all the walls of the perystile has a front of salts in nearly all the perimeter and around 1–1.5 meters from the floor, except for the A5 wall where it is lower (around 0.4 m) and A6 which has not salt formation. It is necessary to emphasize that most of the salts are present as sub-efflorescences (salts crystallized inside the pores which generally are hidden), as can be seen in the Fig. 1c. The external plaster is detached and the accumulation of salts is observable as white spherical particles in the inner areas of the mortar. Efflorescences are also present in minor proportion. In summary, the A1-A5 walls are affected by sub-efflorescences or, at least, efflorescences, being the A6 wall the only one not affected by salts formation.

Regarding the wall paintings, they are quite degraded despite of their recently restoration due to the releasing of the different plaster layers promoted probably by the formation of inner salts. Due to this damage, apart from the sub-efflorescences, it is possible to observe the intonaco (the most external and thinnest plaster layer of the frescoes) in some of the walls (see Fig. 1c), which is characterized by the presence of, at least, two different plaster layers: a deeper based on lime and pozzolan, and another one, based on lime and spathic calcite from local rocks^1^.

### *In-situ* measurements by portable Raman spectroscopy

As has been mentioned, the measurements were carried out *in-situ* without take any sample. The field studies were performed in October 2015 during the APUV 2015 expedition. This *in-situ* molecular characterization of the observed salts was performed by means of Raman spectroscopy using a hand-held innoRam ultramobile spectrometer (B_&_WTEK_INC_, Newark, USA) provided with a 785 nm excitation laser with a nominal power of 245 mW and a CCD detector (Peltier cooled). The microprobe was handled manually and spotted directly on the surface of the studied walls around the front of salts. The measurements were carried out in a continuous scan mode in order to measure the whole affected areas. That is, a complete screening of all studied area was carried out obtaining more representative results than doing the classical point-by-point analysis where relevant points would be neglected. When an optimum response was obtained, the measurement point was fixed to improve, collect and save the spectrum observed in the continuous scan mode. To improve them, the integration times, as well as the spectra accumulations were set to obtain the better signal-to-noise ratio. In this way, more than 125 spectra were saved from the continuous scan of the six analyzed walls. All these spectra were obtained with a spectral resolution of 3 cm^−1^ in a spectral range of 125–2500 cm^−1^. In all cases the laser power was reduced below 10% of the nominal maximum power to avoid photodecomposition and/or chemical transformation of the analysed compounds. Data acquisition was done with BWSpec^TM^ 3.26 software version (B_&_WTEK_INC_, Newark, USA), after a daily calibration with a silicon chip using the 520 cm^−1^ Raman line. The analysis, treatment and interpretation of the results were realized with Omnic Nicolet software. The interpretation of Raman results was accomplished by comparison with standard Raman spectra from own databases as e-Visart and e-Visarch^17,18^ and spectra obtained from on-line databases such as RRUFF^19,20^.

## Results and Discussion

**Figure 2:**
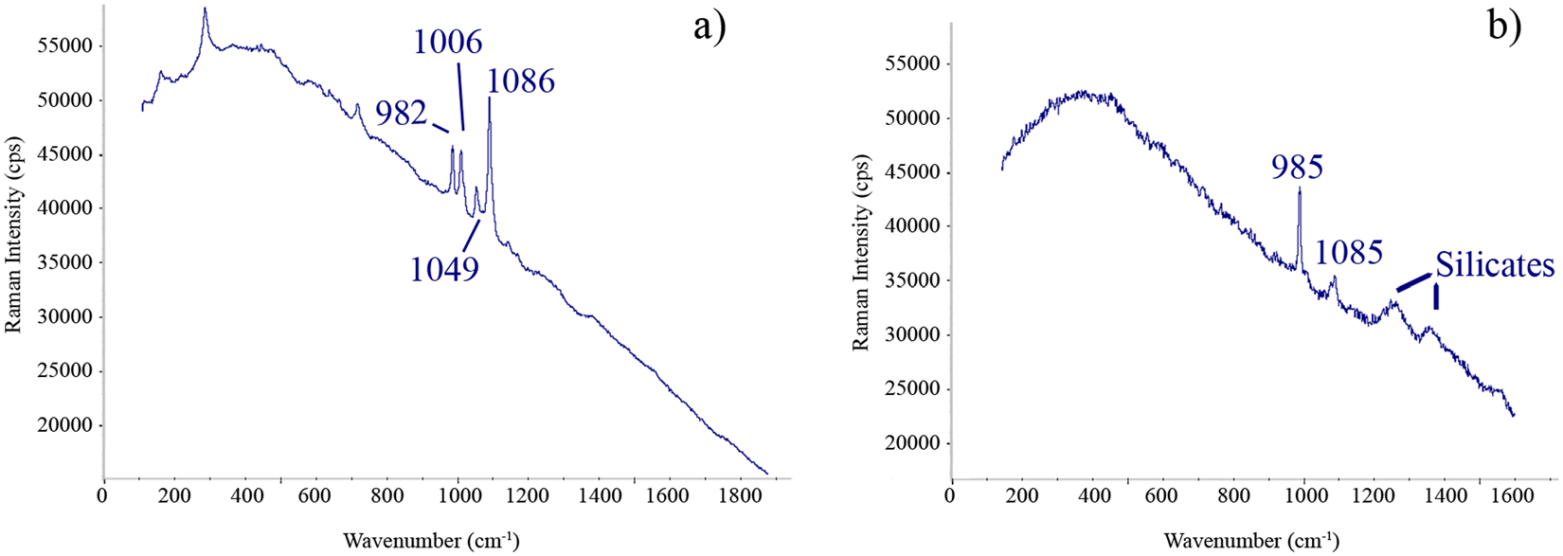
Two Raman spectra collected in the A1 wall of the peristyle in House of Gilded Cupid (Pompeii): (**a**) Calcite (1086 cm^−1^), syngenite (982 and 1006 cm^−1^) and niter (1049 cm^−1^) were identified as a complex mixture, (**b**) epsomite (985 cm^−1^) and calcite (1085 cm^−1^) were observed together with fluorescence signals from silicates.

The walls of the A1 area are quite affected by sub-efflorescences which can be observed in the detachment of the most external layer of the plaster. After a carefully identification of the complex mixture of salts, these walls revealed the presence of calcite (CaCO_3_, bands at 155, 282, 713, 1086, 1436 and 1749 cm^−1^), gypsum (CaSO_4_·2H_2_O, bands at 185, 416, 490, 672, 1008 and 1134 cm^−1^ Raman bands), thenardite (Na_2_SO_4_, bands observed at 993, 1131 and 1152 cm^−1^), syngenite (K_2_Ca(SO_4_)_2_·H_2_O, bands at 443, 633, 644, 662, 982, 1006, 1139 and 1169 cm^−1^), epsomite (MgSO_4_·7H_2_O, main band at 985 cm^−1^), nitratine (NaNO_3_, bands at 722 and 1067 cm^−1^) and niter (KNO_3_, identified Raman bands at 715 and 1049 cm^−1^). Figure 2 shows Raman spectra for some of the described salts. It is necessary to remark that the bands given are related to the best spectra collected in the corresponding wall, for that reason the bands given in each walls could be different in the number of observed bands. Moreover, the presence of a compound is verified if five clear and good Raman spectra are obtained in the studied area.

The identification of nitrates must be highlighted because their presence is important due to its high solubility and therefore, their damaging power. Apart from clearly characterized nitrates due to specific Raman signals in most of the spectra taken in this A1 area, a small and wide band at 1050–1053 cm^−1^ frequently appears which is also related to nitrates^21^. This fact is a usual problem of the *in-situ* Raman analyses due to the low intense (medium/weak) bands which are difficult to be clearly detected, as in the case of nitrates. However, in this area, the presence of sodium and potassium nitrates were clearly identified in most of the *in-situ* analysis by the presence of their secondary Raman bands.

The presence of nitrate salts in the A1 wall requires an important source of dissolved nitrate. In the back side of this A1 wall there is an open garden belonging to the neighbor house. A recent work^22^ has shown the important nitrate concentrations that present natural soils covered by grass in a non-polluted area (around 40 mg/kg of water extractable nitrate); in that work, the decomposition of the grass was claimed as the source of nitrates in the form of ammonium nitrate (NH_4_NO_3_). Thus, infiltration waters from rainfalls washing the adjacent garden are the source of nitrate supply; being the accompanying ammonium the acid that attacks the alkaline carbonates as in the equation (1) (M: sodium, potassium or similar)^23^:

1 $${{\rm{M}}}_{2}{{\rm{CO}}}_{3}+2{{{\rm{NH}}}_{4}}^{+}+2{{{\rm{NO}}}_{3}}^{-}\to 2{{\rm{MNO}}}_{3}+{{\rm{CO}}}_{2}({\rm{g}})+{{\rm{NH}}}_{3}({\rm{g}})+{{\rm{H}}}_{2}{\rm{O}}$$

Original compounds of building materials were also identified such as hematite (α-Fe_2_O_3_, observed Raman bands at 290 and 410 cm^−1^), sanidine (K(AlSi_3_O_8_), main band at 514 cm^−1^) and other silicates. Some silicates present in the analyzed plasters, such as sanidine are commonly present in volcanic areas and, in particular, in the Vesuvius surroundings^24^.

The analysis of the A2 wall revealed less variety of salts respect to A1. The most common compound identified in the (sub-)efflorescences was calcite. This identification was clearly related to the salts accumulation, and not to an original component of the plaster, since the size of the formed salt aggregates is large enough to avoid the measurement of the original composition of the mortar. The source of this salt could be due to the acid attack to the original calcite of the mortar that first dissolve and then transport the Ca^2+^ and HCO_3_^−^ ions, until a subsequent reprecipitation occurs when the water evaporates^25^. This reaction is widely described elsewhere^26–28^ and it is one of the most important problems of calcite based construction materials even nowadays.

In the A2 wall gypsum, aphthitalite ((K,Na)_3_Na(SO_4_)_2_, bands at 452, 621, 629, 993, 1084 and 1202 cm^−1^) and syngenite were also identified in less amount than in the A1 wall. In this case also, the band at 1050–1053 cm^−1^ related to nitrates was also observed in some of the spectra, increasing the number of spectra in which they were present in sampling zones towards the A3 wall (south orientation).

**Figure 3:**
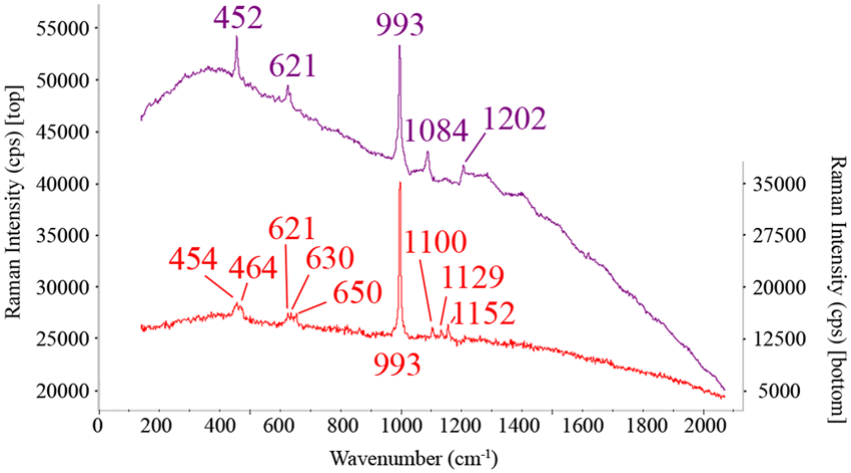
Raman spectra of aphthitalite (top) obtained in the A2 wall and thenardite (bottom) from the A4 wall in the peristyle.

Special attention should be paid to the assignation of the band at 993 cm^−1^. This strong band can be assigned to aphthitalite or thenardite. Moreover, both sulphates have a similar spectral signature at low frequencies (in Fig. 3 an example of the spectra of both compounds obtained in different walls can be observed). Their correct identification is important because they do not have the same damage capacity (in addition to the solubility thenardite forms a crystallization cycle with mirabilite at easily achieve temperatures and humidity levels which involves an important volume change promoting stress inside materials) and therefore, the relevance for the assessment of the conservation state of the Pompeian houses is not the same^9,29^. In this sense, aphthitalite was identified in the spectra thanks to the intensity ratio between the 452 and 993 cm^−1^ bands, which is different for thenardite^30^ (see Fig. 3). In addition, the spectra of both sulphates at high frequencies of the main 993 cm^−1^ band are different as can be seen in the Fig. 3 thus, the secondary bands at 1084 and 1202 cm^−1^ are only present in the aphthitalite spectrum, whereas the 1100, 1129 and 1152 cm^−1^ ones are present in the thenardite compound^30^. Taking all these into account, the presence of thenardite was discarded in this A2 wall because these secondary bands of thenardite were not observed.

Finally, the last salt identified in the A2 wall was weddellite (CaC_2_O_4_·2H_2_O, bands at 190, 907 and 1473 cm^−1^). This salt could indicate the presence of bioactivity, because this compound is related to microorganism metabolic excretions, which can be harmful for the building materials^31^. Regarding other types of identified compounds different from salts, sanidine was again identified like in the A1 wall.

**Figure 4:**
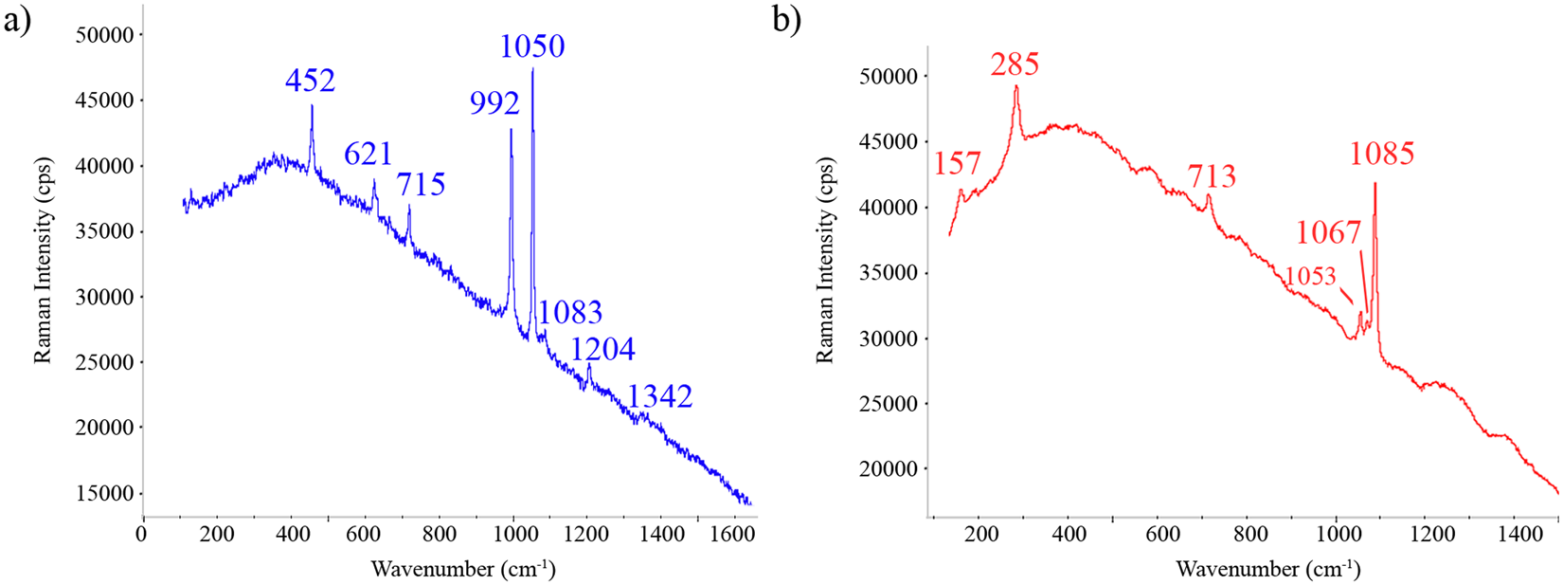
Raman spectrum obtained from A3 wall of the peristyle: calcite (156, 280, 715 and 1086 cm^−1^), sanidine (514 cm^−1^), niter (1050 cm^−1^) and silicates with wide and intense fluorescence bands located at higher wavenumbers than 1200 cm^−1^.

The A3 wall presented more affection by salts than the previous areas, not only because of the presence of a complex mixture of salts, but also for the observed amount of (sub)efflorescences. In this case, calcite was not so easily identified as a salt, being different sulphates the most common identified salts. For example, thenardite (Na_2_SO_4_, bands at 454, 623, 632, 650, 993, 1100, 1129 and 1152 cm^−1^) was identified. Moreover, nitrates were also observed in this wall, as can be seen in the Fig. 4 where niter is observed; niter and nitrocalcite have the same maximum wavenumber, but can be differentiated due to their different FWHM (Full Wide at Half Maximum) values, FWHM = 8 cm^−1^ for KNO_3_ and FWHM = 22 cm^−1^ for Ca(NO_3_)_2_.6H_2_O^32,33^. However, as in the A2 wall, all these salts were present more as efflorescences on the wall than as internal salts, thus, the deterioration level of the mortar was not so notable. Finally, silicates like sanidine were observed, once again, as original composition of the mortars.

**Figure 5:**
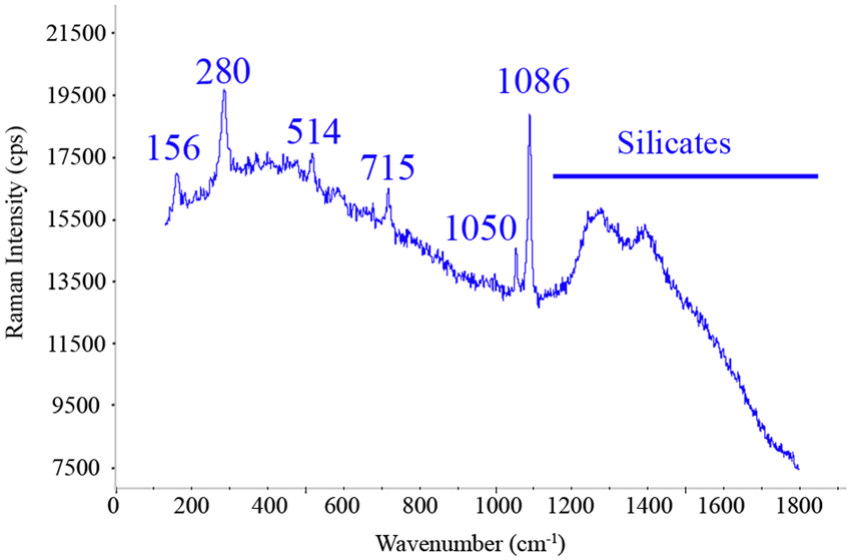
Raman spectra of the A4 wall of the peristyle. (**a**) Niter (715, 1050, 1342 and 1359 cm^−1^) together with aphthitalite (452, 621, 992, 1083 and 1204 cm^−1^). (**b**) Nitratine (1067 cm^−1^), calcite (157, 285, 713 and 1085 cm^−1^) and the complex nitrate signature (1053 cm^−1^).

The A4 wall was severely affected by the salts formation as subefflorescences even more than the case of the A1 wall. In this area, it was possible to observe detachments of the recently restored mortar and the presence of white spherical particles of salts inside it (see Fig. 1c). According to the chemical composition of these balls, the salts present were similar to the salts identified in the A1 area, except epsomite, which was not observed in this case. However, in this area it were identified again potassium and sodium nitrates without ambiguity despite the complexity of the mixture of salts, as well as, the band of unknown nitrates. Figure 5 shows two complex Raman spectra collected in this A4 area.

The analysis of A5 wall revealed a very low affection and in general, the most common salt observed was calcite. The 992 cm^−1^ band related to aphthitalite or thenardite was observed, as well as the wide band at 1050–1053 cm^−1^ of mixed nitrates. Unfortunately, in both cases, the spectra had not a high signal-to-noise ratio to identify the real nature of these compounds due to the absence of significant secondary Raman signals in most of the collected spectra in this area. Moreover, the height of the salt front was quite lower than in the other cases.

The A6 wall was investigated for original mineral phases because it did not shown decayed salts. Again calcite, sanidine and silicate materials (puzzolans) were detected.

It is necessary to remark that the collected spectra were carried out in a continuous scan in order to obtain a representative data of the whole peristyle, avoiding the point-by-point analysis. In this sense, the identified compounds are not the results of a point-by-point measurement but a map of the whole studied area, being representative of composition of the observed efflorescences.

The results obtained from each wall were compared taking into consideration the orientation of each studied wall (see Table 1). However, there was not an evident contribution of the rainwater in the relative presence of salts, as it was expected. Thanks to the information collected from the Italian National Institute for Environmental Protection and Research (ISPRA) it was possible to determine a general pathway in the prevalence of the low winds in Pompeii^7,34–36^. The prevalent winds of Pompeii are from east-northeast (ENE) and south-southwest (SSW), being both directions similar in percentage and speed^34–36^. Taking this data into account, the most affected wall by the rainfalls and therefore, by soluble salts would be A4, which has the wall paintings directly oriented to rainfalls from the ENE and the wall A6, which is oriented to the SSW winds and rains. However, it was observed that the A4 is most affected by soluble salts but the A6 it is not affected by them. Thus, the affection seemed to be not conditioned only by direct impact of the rainfall in the wall paintings.

Observing the roofs distribution (Fig. 1a and Table 1) a link between the level of protection by the use of ceilings and the salt damage was noticed. For instance, the A5 and A6 areas were the most protected ones, with ceilings in both sides of the wall, and were the less affected by salts. Bearing in mind this observation, the most affected walls (A1, A3 and A4 areas) correspond to those walls where the backside, belonging to a garden and another room of the neighbor house, are not protected by ceilings (or roofs) and are exposed directly (or almost directly) to the prevalent winds and rains of Pompeii. In the same way, the A2 wall that it is not affected by the direct impact of the rainfall is the less affected among the walls without protection in its backside. And finally, as it has been previously mentioned, the walls protected by both sides (A5 and A6 walls) do not present remarkable affections. In this last case, and particularly in the A5 area, some affection by soluble salts was detected. In this wall, the observed front of salts is located at less height from the floor than in the other walls. Moreover, in this area and in rainy days an accumulation of water because of the irregular floor is usually observed, thus the formation of the salts in this area seems to be correlated with this punctual phenomenon that occurs when it rains hardly, probably because of the absorption of the accumulated rainwater by capillarity.

**Table 1:** Summary of the different compounds identified in the different walls of the peristyle in the House of Gilded Cupid (Pompeii), the orientation of the non-protected backside of the measured wall and the level of affection by salts calculated by the predominance of salts in the measurements (n.a: not applicable).

	Compound	Formula	A1	A2	A3	A4	A5	A6
SALTS	Calcite	CaCO_3_	X	X	X	X	X	
	Gypsum	CaSO_4_·2H_2_O	X	X	X	X		
	Aphthitalite	(K,Na)_3_Na(SO_4_)_2_		X	X	X		
	Thenardite	Na_2_SO_4_	X		X	X		
	Syngenite	K_2_Ca(SO_4_)_2_·H_2_O	X	X	X	X		
	Epsomite	MgSO_4_·7H_2_O	X					
	Nitratine	NaNO_3_	X			X		
	Niter	KNO_3_	X			X		
	Weddellite	CaC_2_O_4_·2H_2_O		X				
	Thenar. or aphth.	NaSO_4_ or (K,Na)_3_Na(SO_4_)_2_					X	
	Undetermined nitrates	(Ca,K,…)NO_3_	X	X	X	X	X	
	Sanidine	KAlSi_3_O_8_	X	X	X			X
	Hematite	Fe_2_O_3_	X					
	Undetermined silicates	-SiO_x_ (puzzolans)	X		X			X
	Orientation of the wall paintings (protected by roof)		WSW	NNW	NNE	ENE	SSE	SSW
	Orientation of the non-protected backside wall		ENE	SSE	SSW	WSW	n.a	n.a
	Level of affection by salts		+++	+	++	++++	−	n.a

Once the formation of salts have been correlated with the orientation of the non-protected backside of the walls, it has been possible to understand the formation mechanisms. The rainfalls, which impact in the backside of the wall painting, allow the penetration of water (that contains salts and acids) in the walls through the pores of the materials. First, the water of the rain can generate the hydration of the oxides present in the building materials forming more reactive compounds such as hydroxides^21^. Then, these compounds can be attacked by the ions of the rainwater forming the identified sulphates and nitrates^23^. Moreover, the rainwater can also contain other salts like sodium chloride, magnesium sulfate or sodium nitrate (coming from the diffuse impact of marine aerosol around Pompeii) that can be transported inside the pores of the walls, increasing the reactivity with other ions present inside the porous net and precipitating new salts when the water evaporates, leading to the observation of a high variety of efflorescence and/or sub-efflorescence salts^23,33^, as we have observed in this study.

Moreover, the identification of calcite in form of (sub)efflorescence involves the attack of either the atmospheric acid gases transported by rainfalls or the ammonium ion transported by infiltration waters, coming from the garden, because a first acidification step is required to dissolve the original calcite^23^. Thus, the acidic atmosphere around Pompeii seems to have an important role in the decaying processes of the wall paintings in the houses of the archaeological city, together with the infiltration waters that wash the gardens behind the degraded walls supplying the harmful ammonium nitrate.

The studied wall paintings are protected from the wash of the raining so, the loss of material by the rainwater is not so relevant like in walls directly exposed to the open air^7^. However, this fact could be even more harmful than the rainfall wash because the salts remain more time inside the pores of the materials and, generally, when wetting by the infiltration or waters coming from the backside of the walls the reactivity among the different ions increases, leading to the formation of complex mixtures of salts and to the subsequent degradation of materials, in relative short times after the restoration of the protected frescoes, due to the physical stress induced by the solubilization/reprecipitation cycles inside the pores.

## Conclusions

This work evidences the need of portable Raman spectrometers to perform a conclusive and representative identification of the nature of the salts formed on ancient Roman mortars and thus, on other kind of building materials giving the keys to its correct use. Besides, it was even possible to correlate the formation of such salts with the environmental stressors and identify the main factors for their formation. Thus, this work demonstrates how *in-situ* Raman spectroscopy is a useful and easy tool to obtain chemical information that support the relevant decisions that are needed to be taken to preserve the cultural heritage assets. The demonstrated advantages are especially remarkable when the sampling it is not allow. All of these, point out Raman spectroscopy to be implemented as conservation tool, go beyond the academic and research world.

In this sense, the most important reason for the severe affection of salts found in the perystile of the House of Gilded Cupids seems to be related to the rainfall impact in the non-protected backside of some wall paintings. Previous works pointed the need of protect the wall paintings^7^, however, in this work it has been observed that this action is not enough for the safety of the frescoes and the protection of both sides of the walls seems to be crucial. Moreover, in a lower level of severity, the correlation between the salts formation and the water infiltration by capillarity was also observed. Thus, the well protected wall paintings present salt affection only when the rain water is guided to them due to the unevenness of the floor.

On the whole, the present work reveals aspects of paramount importance to ensure the optimal conservation of Pompeii mortars and wall paintings. On the basis of the collected results, our recommendation in order to avoid the wetting of the mural structure by rainfall events or by infiltration waters from the surrounding gardens is to place a roof not only on the side of the walls presenting frescoes and mortars but also their backside.

Considering that the importance of this aspect is often underestimated during the planning of structural interventions, the authorities should take this recommendation into account to improve the conservation of Pompeian remains and avoid the cyclical reappearance of degradation phenomena.

### Data availability statement

The most of the data generated during this study is included in this article, anyway, the other data generated are available from the corresponding author on reasonable request.
